# Health and demographic surveillance systems: contributing to an understanding of the dynamics in migration and health

**DOI:** 10.3402/gha.v6i0.21496

**Published:** 2013-07-11

**Authors:** Annette Gerritsen, Philippe Bocquier, Michael White, Cheikh Mbacké, Nurul Alam, Donatien Beguy, Frank Odhiambo, Charfudin Sacoor, Ho Dang Phuc, Sureeporn Punpuing, Mark A. Collinson

**Affiliations:** 1 Medical Research Council/Wits Rural Public Health and Health Transitions Research Unit (Agincourt), School of Public Health, University of the Witwatersrand, Acornhoek, South Africa; 2 INDEPTH Network, Accra, Ghana; 3 Centre de Recherche en Démographie et Sociétés, Université Catholique de Louvain, Louvain-la-Neuve, Belgium; 4 Population Studies and Training Center, Brown University, Providence, RI, USA; 5 Independent Consultant, Senegal; 6 Matlab HDSS, Centre for Population Urbanization & Climate Change, icddr, b, Bangladesh; 7 African Population and Health Research Centre, Nairobi, Kenya; 8 KEMRI/CDC Research and Public Health Collaboration, Kisumu, Kenya; 9 Manhiça HDSS, Mozambique; 10 Filabavi HDSS and Institute of Mathematics (VAST), Hanoi, Vietnam; 11 Kanchanaburi HDSS, Thailand; 12 Mahidol University, Nakhon Pathom, Thailand; 13 Centre for Global Health Research, Umeå University, Sweden

**Keywords:** migration, health and demographic surveillance system, determinants, mortality, event history analysis

## Abstract

**Background:**

Migration is difficult to measure because it is highly repeatable. Health and Demographic Surveillance Systems (HDSSs) provide a unique opportunity to study migration as multiple episodes of migration are captured over time. A conceptual framework is needed to show the public health implications of migration.

**Objective/design:**

Research conducted in seven HDSS centres [International Network for the Demographic Evaluation of Populations and Their Health (INDEPTH) Network], published in a peer-reviewed volume in 2009, is summarised focussing on the age–sex profile of migrants, the relation between migration and livelihoods, and the impact of migration on health. This illustrates the conceptual structure of the implications of migration. The next phase is described, the Multi-centre Analysis of the Dynamics In Migration And Health (MADIMAH) project, consisting of workshops focussed on preparing data and conducting the analyses for comparative studies amongst HDSS centres in Africa and Asia. The focus here is on the (standardisation of) determinants of migration and the impact of migration on adult mortality.

**Results:**

The findings in the volume showed a relatively regular age structure for migration among all HDSS centres. Furthermore, migration generally contributes to improved living conditions at the place of origin. However, there are potential negative consequences of migration on health. It was concluded that there is a need to compare results from multiple centres using uniform covariate definitions as well as longitudinal analysis techniques. This was the starting point for the on-going MADIMAH initiative, which has increased capacity at the participating HDSS centres to produce the required datasets and conduct the analyses.

**Conclusions:**

HDSS centres brought together within INDEPTH Network have already provided strong evidence of the potential negative consequences of migration on health, which contrast with the beneficial impacts of migration on livelihoods. Future comparative evidence using standardised tools will help design policies for mitigating the negative effects, and enhancing the positive effects, of migration on health.

A migrant is a person who has established a new place of usual residence (e.g. locality, district) other than the one in which they habitually lived (1). International migration is a change of residence involving the spatial movement of persons across country borders while internal migration is a residence change within the same country (e.g. rural to urban) (1). In this article, the focus is largely on the latter. It was estimated that globally there were 740 million internal migrants in 2009, and there are widespread country differences reported with respect to the intensity of internal migration (2). The reasons for migration can be economic, social, political, or environmental.

Migration impacts on the place and household members left behind as well as on the place where migrants settle, in addition to creating a link between these two places among which people, information, and goods may flow. Migration is related to human capital (housing and family transitions) as well as to labour market conditions (economic opportunities at destination) (3). Remittances from migrants can change the socio-economic status (SES) of households left behind by the migrant (4). Furthermore, there is a bi-directional connection between migration and health status: migration may influence health outcomes, and a person’s health may affect one’s decision to migrate or the destination one seeks (5, 6).

With respect to the effects of migration on health, migration can induce stress (health outcome) and in addition stress can be related to other health outcomes (e.g. reduced immunity) (3). Migration might have an effect on fertility, e.g. by stopping or reducing sexual intercourse between partners or on the contrary by exposing migrants to unprotected sex (7). The living circumstances of migrants might negatively impact health outcomes, e.g. when migrants settle in insalubrious shanty towns (8). Furthermore, movement to a different environment, e.g. an urban area with a different lifestyle and food consumption pattern, a higher elevation or a different climatic zone, can dramatically reduce or increase exposure to diseases (9). In addition, migrants with health knowledge specific to the place they stayed in originally may be less prepared to take protective measures in their new location, and may experience difficulties in accessing available services (10).

Looking at the effects of health on migration, individuals and households may migrate, e.g. from a rural to an urban area to gain access to treatment for a specific condition or in general to be in a place where the overall availability and quality of health services are higher (11). Conversely, individuals, especially in resource-poor settings, may move back from the urban to the rural area to be nearer to family and other caretakers when they are sick, despite insufficient health infrastructure in their place of origin (12, 13). Furthermore, in general, healthy individuals (e.g. younger individuals) are more likely to undertake migration, and age is a major factor in migration selection: the healthy migrant effect (1, 14).

Migration is difficult to measure notably because it is highly repeatable (multiple moves per individual over a period of time). Migration research is largely based on data from censuses and surveys (15). While censuses and Demographic and Health Surveys (DHSs) are usually conducted nationwide they collect data on a cross-sectional basis at generally long intervals (e.g. 10 years for censuses, 5 years for DHSs) and include limited information on migration (e.g. place of birth, place of residence in a prior year, or place of previous residence). This results in an underestimation of the number of migrations and circular and other temporary migration patterns cannot be discriminated (16). This data can be supplemented by Health and Demographic Surveillance Systems (HDSSs) which can track the migration dynamics using a prospective, longitudinal approach. HDSS centres monitor demographic and health characteristics of a population living in a well-defined geographic area (16). HDSS centres do not include nationally representative samples, but they collect data exhaustively, prospectively, and longitudinally, including information on migration (16). Standardisation of data collection and analytic procedures are promoted by the International Network for the Demographic Evaluation of Populations and Their Health (INDEPTH), an umbrella organisation for a network of independent HDSS centres in low- and middle-income countries (17). INDEPTH provides the opportunity to collate data from different centres into outputs that enable systematic comparisons between various contexts (17). However, up until recently, cross-centre comparison of migration data was challenged by the lack of a standard definition of migration in HDSS centres, e.g. varying threshold cut-offs used for defining migration (from 1 to 6 months).

The conceptualisation of migration from a public health perspective can be challenging due to measurement difficulties and contradictory evidence. This article describes the conceptual framework used by the Migration, Urbanisation and Health Working Group of the INDEPTH Network when producing a peer-reviewed volume in 2009 on migration, livelihoods, and health. This led to a more systematic examination of these issues by means of more rigorous multi-centre comparative studies, which is also described. Examples are used from the volume to illustrate key concepts in the framework.

## The dynamics of migration, health and livelihoods – INDEPTH Network perspectives

The data presented in the INDEPTH volume with this title, published in 2009, show features of the positive and negative effects of migration from a public health perspective (18). The research questions in the book, which reflect the structure of the conceptual framework, are: (1) What is the age–sex profile of migrants? (2) What is the relation between migration and livelihoods? (3) What is the impact of migration on morbidity and mortality? The answer to the first question gives information on who migrates. The second question relates to whether the most common goal of migration, namely economic benefits, is reached. The last question relates to the positive or negative consequences which migration might have on the health of migrants as well as their children. In eight chapters, the volume brings together work that has been done within seven individual HDSS centres based in Africa and Asia that are part of the INDEPTH Network. In these chapters migrants are mainly internal migrants, unless specifically stated otherwise.

### What is the age–sex profile of migrants?

Despite the diversity of contexts from which the data originated (rural and urban, African and Asian) and some variation in the threshold of time used to classify migration, a relatively regular age structure for migration was found (19). The largest group are young adults (both males and females, aged 20–24 years), sometimes accompanied by children (especially under 5 years). The age patterns indicate that key components of these migration profiles are labour migration, children’s migrating with parents, and, to a lesser extent, marriage formation or dissolution or households moving to access better services. Migration at older ages reflects movements associated with seeking health care or services or exit from the labour force. Cycles of returning in-migration are more pronounced for males than females, and the age profile of returning migration varies by setting.

### What is the relation between migration and livelihoods?

In Matlab (rural Bangladesh), findings on the association between parent’s migration and children’s education (20) indicated that (mostly international) male migrants ensure better educational outcomes to their children remaining at home than non-migrants. More specifically, the results from a regression analysis showed a higher expected rate of school enrolment and progression from grades 5, 7, and 10 for children whose fathers were migrants. Long-term and long-distance migration (probably linked to movement to oil-rich middle-east countries) exhibited a stronger effect on education than more proximate migration. These effects persisted even after controlling for father’s and mother’s educational attainment. Migration of mothers was not related to children’s education.

A study conducted in Agincourt (rural South Africa) looked at the relationship between migration and household SES, especially focussing on the patterns and trends in remittances over the period 2000–2007 and the change in household SES (21). It was found that remittances from temporary migrants can help lift families out of poverty. The results of a regression analysis showed that an above-poverty level of living (above the median of an asset-based ‘absolute SES’ indicator) is associated with one or more temporary migrants having circulated from the household to other communities and urban areas. Migrants to big cities are not necessarily the main generators of household of origin’s SES. This effect depends on the employment changes achieved through the migration. Furthermore, it was found that female temporary migrants are important contributors to improved conditions for sending households. Not only do migrants send money, but also clothes and food, and these latter types of remittances are greater from employed female migrants than their male counterparts.

A study conducted in Kanchanaburi (rural Thailand) examined how migration away from agricultural households is associated with changes in the amount of land used in cultivation and the amount of household labour employed in agriculture (22). It was found that the net effect of sending a migrant away from an agricultural household is mixed: it depends on the household’s ability to compensate for this loss through labour reallocation and land-use adjustments. Out-migration results in a reduction in the amount of land used for agriculture. However, agricultural households with more resources, that is, more land under cultivation, are better able to fund one or more members to migrate. Furthermore, households appear to adjust human resources to compensate for the loss of labour through migration.

In summary, these three studies have shown that migration, specifically out-migration from the origin HDSS community, generally contributes to improved living conditions at the place of origin.

### What is the impact of migration on morbidity and mortality?

A study in FilaBavi (rural Vietnam) looked at the relation between migration and child morbidity, focussing on the association between migration and under-five morbidity (23). The regression analysis examined the predictors of sickness episodes by migration status and showed that the burden of childhood morbidity is dependent on the sex of the migrating parent. There was a higher incidence of illness among children left behind by their mothers compared to children who migrated with their mothers. No such negative impact on children’s health of father’s out-migration was observed, underscoring the critical importance of the mother’s role in providing health care to young children.

A study conducted in the informal settlements of Nairobi (urban Kenya) focussed on the effect of mother’s migration on childhood mortality (24). Children born in the slums to migrant mothers exhibit a 1.8 times higher risk of mortality than those born to non-migrant mothers, after controlling for demographic and socio-economic factors. One reason for this finding could be that newly resident mothers and their newborn children have not yet adapted to the environment of the urban slum and hence are perhaps unaware or uncertain how to seek health care services for their children.

On the other hand, a study conducted in Nyanza (rural Kenya) showed that urban–rural child migration was significantly associated with a lower risk of death (25). Children moving from Kenyan urban areas to rural Nyanza exhibited a significant 29% survival advantage, after controlling for household SES and mother’s and father’s education and employment. Children migrating from other rural areas did not have a significantly different survival chance compared to non-migrant children. So, child migration is not necessarily negative and may even be positive if the risks incurred by migration are offset by the health endowments and migrant selectivity associated with the migration.

Historically, returning migrants were expected to be positively selected for health and economic situation, retiring with their earnings to the comfort of their home village and having a lower mortality than non-migrants. However, HIV/AIDS changed the situation, e.g. in South Africa, and migrants returning home could now be in poor health, needing care, and support from their families (12, 13). A study conducted in Manhiça (rural Mozambique) focussed on this reversal of survival advantages of migrants (including to South Africa) aged 20–59 years returning to their home communities using event history analysis techniques. It showed that the reversal occurred around 1999, more than a decade after the emergence of HIV/AIDS (26). Mortality rates increased over the study period for returning migrants as well as for non-migrants, but the increase was steeper for the returning migrants and, from 1999, the mortality became higher for migrants than for non-migrants. Also, a strong association was found between recent return migration and mortality, primarily due to HIV/AIDS. The influx of sick and dying migrants shifted the burden of disease in the rural place of origin and placed heavy stress on the households (including loss of income) and on the local health care systems.

Thus, the findings in the studies focussing on migration and morbidity/mortality highlight the potential negative consequences of migration on health which contrast with the beneficial impacts of migration on livelihoods.

Although the book chapters give answers to the research questions posed, the results are from individual HDSS centres, addressing slightly different objectives, and using different definitions and analytic methods. The book concluded on the necessity to compare results from multiple centres using uniform covariate definitions as well as longitudinal analysis techniques. This was the starting point for the Multi-centre Analysis of the Dynamics In Migration And Health (MADIMAH) initiative.

## Multi-centre Analysis of the Dynamics In Migration And Health

The aims of the INDEPTH MADIMAH project are to: (1) standardise data on migration; (2) standardise techniques used to analyse migration and related aspects; and (3) publish multi-centre comparative analyses. Improved capacity was needed at the HDSS centres level to produce the required datasets and conduct the analyses. Therefore, central in achieving these aims is capacity building of the data scientists and analysts in each participating centre.

The MADIMAH project focuses initially on the following two research questions: (1) What are the determinants of migration? (2) What is the impact of migration on mortality? Note that in the future, research questions regarding the association between migration and fertility on one hand, and migration and SES on the other might be addressed and therefore some preparatory work on those aspects is included in the project activities.

Establishing the determinants of migration is important for two reasons. Firstly, migration is a key socio-demographic event, and it is therefore important to identify its determinants at community, household, and individual levels. This has not been done systematically so far in HDSS centres, i.e. using the same definition of migration and determinants as well as the same analytical techniques. Secondly, if the impact of migration on mortality needs to be estimated, it is important to know the factors related to migration before to the move, because these may also be related to the risk of mortality.

It is important to study the relation between migration and mortality for three main reasons. Mortality is a major indicator of health and one that is measured in all HDSS centres. Secondly, migration could be dependent on health status with the latter either impeding or stimulating migration. Thirdly, migration is usually conducted for economic benefits, which – as was seen in the book chapters and other publications (12, 13) – can show simultaneously negative health outcomes and place extra burdens on households and the health system.

The MADIMAH project consists of a series of workshops focussed on preparing the data for a comparative analysis amongst HDSS centres in Africa and Asia to answer the posed research questions as well as to conduct the analysis. Training materials are developed and presented by experienced statisticians who have worked with data from multiple centres (co-authors PB, DB, and MC). These are used during the workshops and later on adapted (if needed) for future use in workshops or on-site guidelines for centres. In-between workshops, centres are assisted from a distance to finalise the research outputs.

### Dataset preparation and preparatory analyses

The first phase focussed on the dataset preparation and preparatory analyses. A 5-day workshop was held in April 2011 in Accra (Ghana) with 13 HDSS centres present. Objectives of the workshop were to: (1) take stock of which data is available from each centre; (2) prepare a residency file for each centre; (3) convert this file to a ‘long’ format that is adequate for event history analysis; (4) check the consistency of dates and sequences of events; (5) learn to set the data for event history analysis; and (6) learn how to graph and tabulate migration and mortality indicators.

In October 2011, a second 3-day workshop was held in Maputo (Mozambique) with 14 HDSS centres. The training focussed on event history analysis (micro-data longitudinal analysis) for migration, mortality, and fertility studies. The workshop outputs included: (1) Data consistency matrices for the initial database and the final database to show data quality improvement before and after quality improvement initiatives. Indicators of data quality include percentage of data records that have been deleted and percentage of inconsistencies remaining, as well as comments on the nature of remaining inconsistencies. (2) Graphs showing the age–sex profile of mortality rates, out-migration rates, and in-migration rates, from onset of data collection to the most recent data collection year.

A third two-and-a-half-day workshop was held with 15 HDSS centres in June 2012 in Accra. The training continued on the topic of event history analysis for mortality and fertility studies. The workshop aims were: (1) checking data for conformity to INDEPTH ‘Core minimum micro-data set specifications’ as required for the INDEPTH Sharing and Access Repository (iShare2); iShare2 aims to enhance the research data management capacity of INDEPTH member centres and expand data sharing by establishing and maintaining a data repository (www.indepth-ishare.org) (17); (2) producing mortality indicators; (3) preparing and organising data for fertility analysis; and (4) producing fertility indicators.

### Analysis of the dynamics in migration and health

In February 2013, a 4-day analytical workshop was held in Nairobi (Kenya) on determinants of migration in 13 HDSS centres. Aims of this workshop were to standardise measures of migration and determinants and to do descriptive and longitudinal regression analyses of determinants of migration. This included the analysis of time-changing covariates such as education and union status (e.g. married and living with spouse, married with spouse living elsewhere, not in union, etc.) using Cox models. This will lead to publications on the determinants of migration by each centre as well as to multi-centre publications.

Another workshop is planned towards the end of 2013 focussing on migration and mortality which will build further on the previous workshops and focus on the analysis of migration and mortality.

The MADIMAH project has enabled efforts in standardisation of data preparation and analysis for studying migration in relation to the SES of the households and the health and wellbeing of migrants, especially in terms of mortality. A present limitation is that the current project cannot see what happens to out-migrants who have left the study population (which is similar to the problem of loss to follow-up in non-migration studies). To obtain this information, the routine of HDSS data collection should be complemented by follow-up surveys in the most common out-migration destinations to cover issues of changes in, e.g., health status and SES. Tests of follow-up through mobile phones 1 year after migration have been successfully conducted in three INDEPTH centres (27).

It has been argued that HDSS centres are relatively expensive and their data take time to be collected and analysed. They are often underutilised in providing evidence for improving the health of the populations studied (16). The latter is often due to lack of sufficient data management and statistical skills on-site (17). Through the workshops described above, the multi-centre project contributes to an increase in resident data management and analysis capacity.

In conclusion, HDSS centres brought together within INDEPTH Network have already provided evidence for understanding the dynamics in migration and health. Further analyses using standardised tools are needed to build comparative evidence. This will potentially lead to adaptation of policies and targeting of interventions for mitigating the negative effects, and enhancing the positive effects, of migration on health.
